# Post-intensive care syndrome follow-up system after hospital discharge: a narrative review

**DOI:** 10.1186/s40560-023-00716-w

**Published:** 2024-01-12

**Authors:** Nobuto Nakanishi, Keibun Liu, Junji Hatakeyama, Akira Kawauchi, Minoru Yoshida, Hidenori Sumita, Kyohei Miyamoto, Kensuke Nakamura

**Affiliations:** 1 Division of Disaster and Emergency Medicine, Department of Surgery Related, Kobe University Graduate School of Medicine, 7-5-2 Kusunoki, Chuo-Ward, Kobe, 650-0017 Japan; 2 Critical Care Research Group, The Prince Charles Hospital, 627 Rode Rd, Chermside, QLD 4032 Australia; 3 Institute for Molecular Bioscience, The University of Queensland, 306 Carmody Rd, St Lucia, QLD 4067 Australia; 4 Non-Profit Organization ICU Collaboration Network (ICON), 2-15-13 Hongo, Bunkyo-Ku, Tokyo, 113-0033 Japan; 5 Department of Emergency and Critical Care Medicine, Osaka Medical and Pharmaceutical University, 2-7 Daigaku-Machi, Takatsuki, Osaka 569-8686 Japan; 6 Department of Critical Care and Emergency Medicine, Japanese Red Cross Maebashi Hospital, 389-1, Asakura-Machi, Maebashi-Shi, Gunma 371-0811 Japan; 7 Department of Emergency and Critical Care Medicine, St. Marianna University School of Medicine, 2-16-1, Sugao, Miyamae-Ku, Kawasaki, Kanagawa 216- 8511 Japan; 8 Clinic Sumita, 305-12, Minamiyamashinden, Ina-Cho, Toyokawa, Aichi 441-0105 Japan; 9 Department of Emergency and Critical Care Medicine, Wakayama Medical University, 811-1 Kimiidera, Wakayama, Wakayama 641-8509 Japan; 10 Department of Critical Care Medicine, Yokohama City University School of Medicine, 3-9 Fukuura, Kanazawaku, Yokohama, 236-0004 Japan

**Keywords:** PICS, Intensive care unit, Assessments, Rehabilitation, Nutrition

## Abstract

**Background:**

Post-intensive care syndrome (PICS) is the long-lasting impairment of physical functions, cognitive functions, and mental health after intensive care. Although a long-term follow-up is essential for the successful management of PICS, few reviews have summarized evidence for the efficacy and management of the PICS follow-up system.

**Main text:**

The PICS follow-up system includes a PICS follow-up clinic, home visitations, telephone or mail follow-ups, and telemedicine. The first PICS follow-up clinic was established in the U.K. in 1993 and its use spread thereafter. There are currently no consistent findings on the efficacy of PICS follow-up clinics. Under recent evidence and recommendations, attendance at a PICS follow-up clinic needs to start within three months after hospital discharge. A multidisciplinary team approach is important for the treatment of PICS from various aspects of impairments, including the nutritional status. We classified face-to-face and telephone-based assessments for a PICS follow-up from recent recommendations. Recent findings on medications, rehabilitation, and nutrition for the treatment of PICS were summarized.

**Conclusions:**

This narrative review aimed to summarize the PICS follow-up system after hospital discharge and provide a comprehensive approach for the prevention and treatment of PICS.

## Introduction

Since advances in critical care have improved survival rates, long-term management has gradually been highlighted to restore the functional capabilities of intensive care unit (ICU) survivors [1]. Post-intensive care syndrome (PICS) is the long-lasting impairment of physical functions, cognitive functions, and mental health after intensive care [1]. A previous study reported that 56% of patients exhibited some impairment in one of the three components of PICS in the 12 months after hospital discharge [2]. Furthermore, 40% of pre-employed ICU survivors were unable to return to work 12 months after hospital discharge [3]. PICS may persist for more than 10 years after discharge [4].

Although numerous bundles are often implemented to prevent PICS, the interventions employed during hospital stays are insufficient to prevent PICS [5, 6]. Therefore, a long-term follow-up is essential for the successful management of PICS [7]. One strategy is a PICS follow-up system with a multi-disciplinary team [8, 9]. A PICS follow-up clinic provides PICS evaluations, follow-ups, and treatments through the expertise of each specialized member, while telephone-based interviews allow for remote follow-ups [9]. There are various ways for this PICS follow-up systems.

Recent systematic reviews and meta-analyses reported the importance of PICS follow-up system [10, 11]. However, few reviews summarized the detail of follow-up system, assessment methods, and contents of management. This information is essential to launch the PICS follow-up system in each facility. Therefore, we summarized the history of and evidence for the PICS follow-up system and its management. In this review, we used the term “PICS follow-up system” as comprehensive system including telephone and online, and the term “PICS follow-up clinic” as the outpatient clinic.

## History of PICS follow-up clinics

PICS follow-up clinics are called “PICS clinics”, “ICU follow-up clinics”, or “ICU recovery centers” [12–14]. The first PICS follow-up clinic, “Intensive after care after intensive care”, started at a London hospital in 1993 [8]. Physicians and nurses in the U.K. began following long-term outcomes after critical illnesses. The international conference “Surviving Intensive Care” held in Brussels in 2002 stated that ICU survivors need to be followed up for more than six months. A PICS follow-up clinic was proposed as an approach to evaluate ICU survivors and their families’ long-term outcomes [15]. In 2006, PICS follow-up clinics were available in 30% of U.K. ICUs [16]. PICS follow-up clinics that are mostly managed by nurses have since increased in the U.K. [17] and Sweden [18].

In the U.S., the first PICS follow-up clinic started in 2011 [19] after the Society of Critical Care Medicine proposed the PICS concept in 2010 [1]. The Society of Critical Care Medicine THRIVE collaboratives, the group working for Post-ICU Clinic and Peer Support, globally provided ICU survivors and their families with education, community, and quality improvement [20, 21]. Furthermore, the National Institute for Health and Care Excellence in 2017 recommended that “Adults who stayed in critical care for more than 4 days and were at risk of morbidity have a review 2 to 3 months after discharge from critical care” [22].

In contrast, PICS follow-up clinics are not common in Japan. In Japan, the first hospital-scale PICS follow-up clinic opened in Ibaraki prefecture at 2019 [12]. The PICS committee of the Japanese Society of Intensive Care Medicine conducted a questionnaire survey in 2021, and reported that PICS follow-up clinics were only conducted in 4% (4/110) of ICUs [23]. Therefore, further recognition of the importance of PICS follow-up clinics is needed.

## PICS follow-up clinic management

### Evidence for the efficacy of PICS follow-up clinics

Scientific evidence for the efficacy of PICS follow-up clinics is limited. A randomized controlled trial on PICS follow-up clinics at three U.K. hospitals found no significant differences in quality of life (QOL) one year after ICU discharge or medical costs [24]. Furthermore, in another randomized controlled trial on ICU follow-up clinics, health-related QOL was not improved in ICU survivors with type 2 diabetes [25]. Therefore, a Cochrane review summarized 4 randomized controlled trials and concluded that PICS follow-up clinic interventions did not provide sufficient evidence on functional impairment outcomes [26]. However, a meta-analysis that summarized 16 PICS follow-up clinic interventions found that physical therapy prevented depression and the reduction in QOL, while psychological interventions improved posttraumatic stress disorder [10]. Although it is based on weak recommendations, Surviving Sepsis Campaign Guidelines 2021 recommends a post-discharge assessment and follow-up of physical function, cognitive function, and mental health in patients with sepsis or septic shock [27]. Differences in recommendations among studies are due to the timing of interventions and their contents. Organized multidisciplinary interventions may change the evidence of PICS follow-up clinics.

### Practice of PICS follow-up systems

The desirable timing for a follow-up has not yet been clarified. Previous studies reported that the PICS follow-up clinic needs to be attended by patients within one to three months after hospital discharge because PICS develops early after hospital discharge [12, 24, 28]. The Society of Critical Care Medicine and Dutch guidelines recommend that high-risk patients need to be screened 2–4 weeks after hospital discharge and followed up at 6–12 weeks after hospital discharge [29, 30]. Physical dysfunction and muscle weakness have been reported even during ICU stays [31], and cognitive dysfunction and mental health issues are also common in the early course of critical illness [32–34]. A follow-up needs to be conducted at multiple times every few months. In a systematic review, it was common to follow-up patients after 3, 6, and 12 months [35]. Long-term follow-ups are also needed because PICS has been reported in more than 50% of ICU survivors 12 months after discharge [36, 37].

The various methods used for a follow-up. Follow-up systems for critically ill patients after discharge from the ICU include PICS follow-up clinics, visitations to the patient’s home or facility, questionnaires posted by mail or in an e-mail to the patient’s home, and telephone or internet-based telemedicine. Although they are important, it is difficult for severely ill ICU survivors to visit PICS follow-up clinics. Therefore, an assessment through a telephone interview or online may be selected. In a previous study, nurse-led home or facility visitation within 8 weeks of hospital discharge reduced the length of readmission days by approximately 6 days [38]. Home visitation may lead to direct interventions. However, home-based interventions require further study because a previous study showed that physical therapist-led 8-week home-based exercise rehabilitation did not contribute to the recovery of physical functions [39]. A questionnaire evaluation via an e-mail has a lower response rate than that posted in the mail, but is 10 times more cost-effective and has fewer missing values [40]. A telephone-based follow-up is often used to screen patients requiring a follow-up [41]. In a previous study, 93% of patients preferred telephone follow-ups over face-to-face follow-ups [42]. Other studies conducted nurse-led monthly phone calls for sepsis survivors 6 months after discharge or a psychologist-led mindfulness program 6 weeks after discharge [43, 44]. The findings obtained showed that neither of the telephone-based interventions improved the QOL of ICU survivors. Internet-based telemedicine is more convenient and time efficient than visitations [45]. A systematic review on the PICS follow-up system revealed that telemedicine models of post-ICU care increased recruitment rates, intervention implementation success rates, and participant retention rates [11]. However, its impact on clinical outcomes warrants further investigation.

It is important to choose the population for ICU follow-up clinics because of limited workforce and time allocation. The target population for ICU follow-up clinics should be the patients with high risk of exhibiting PICS, such as patients with prolonged ICU stays, patients requiring ventilatory management, septic patients, patients with delirium, patients with high severity of illness such as APACHE2 and SOFA scores, women, and elderly patients [12, 25, 36, 46–48]. A 2018 literature review on outpatient ICU follow-up clinics suggested that eligible patients should be on ventilator management for at least 48 h or an ICU stay of at least 3–5 days, but less than 20% of these patients are actually followed up [41]. There is still insufficient evidence for optimal follow-up population and further data accumulation is needed.

**Fig. 1:**
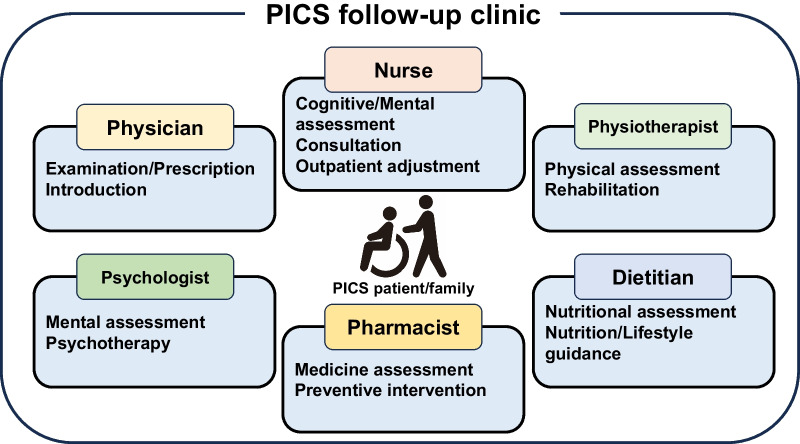
Positions and roles in the PICS follow-up clinic. In the PICS follow-up clinic, physicians, nurses, physiotherapists, dietitians, pharmacists, and psychologists play specialized roles in the management of patients with PICS and their families. The clinic aims to reintegrate PICS patients and their families back into society through the importance of multidisciplinary cooperation

Due to the wide number of PICS symptoms, it is important to provide a multidisciplinary team approach in PICS follow-up clinics with physicians, nurses, physical therapists, pharmacists, clinical psychologists, and dietitians (Fig. 1) [49]. Physicians are responsible for medical examinations, prescriptions, and consultations [43]. Nurses often play a central role in PICS follow-up clinics [12]. Physical therapists evaluate physical functions and provide rehabilitation [9]. Pharmacists contribute to medication management and the identification of adverse drug risks as well as preventive interventions, such as vaccinations [50, 51]. Clinical psychologists evaluate mental health and provide preventive measures for psychiatric symptoms [9, 52]. Dietitians evaluate the nutritional status and provide nutritional education, including oral nutritional supplements and dietary menu arrangements [53].

## PICS assessment instruments

### PICS assessment in PICS follow-up system

The PICS assessment is the initial step in follow-up of ICU survivors, which allows us for a precise evaluation of their conditions and facilitates intervention to enhance their well-being. However, there are too many instruments available for assessing PICS, and as of now, they lack standardization [35, 54]. Previously, various expert groups have recommended distinct sets of tools, including over 30 recommended PICS assessment instruments (Table 1) [29, 35, 55–57]. An appropriate selection is required for different follow-up methods such as face-to-face, telephone, and online questionnaires. We can consider the frequency of usage in previous literature (as shown in Table 1) for the selection process, because instruments that are frequently used are suitable for comparing performance across facilities, and are often deemed valid and user-friendly.

**Table 1:** PICS assessment instruments

Assessment instruments	Items	Score range	Methods	Interview style	Frequency used^a^	Features
Physical functions						
6-min walk test (6MWT)	1	≥ 0	Interview	Face-to-face	49	The value depends on age, sex, body weight, and height
2-min walk test (2MWT)	1	≥ 0	Interview	Face-to-face	3	Short version of 6MWT. The results of 2MWT correlate with those of 6MWT
Medical Research Council score (MRC score)	12	0–60	Interview	Face-to-face	34	Sum of the score of muscle strength by manual muscle strength (0–5) at 12 points
Grip strength	1	≥ 0	Interview	Face-to-face	34	Testing requires a grip dynamometer
Timed Up-and-Go (TUG)	1	≥ 0	Interview	Face-to-face	5	Simple evaluation of mobility that measures how long it takes to stand up, walk a distance of 3 m, walk back, and sit down again
Short Physical Performance Battery (SPPB)	3	0–12	Interview	Face-to-face	14	Evaluation of balance, walking, and sit-to-stand
30-s sit-to-stand (30STS)	1	≥ 0	Interview	Face-to-face	15	Evaluation of lower extremity strength that measures the number of times a patient fully stands up from and sits down a chair without arm muscles in 30 s
Clinical Frailty Scale (CFS)	1	1–9 (ordinal)	Interview	Face-to-face/Telephone	16	Evaluation of the degree of disability from frailty
Cognitive functions						
Montreal Cognitive Assessment (MoCA)	8	0–30	Interview	Face-to-face/Telephone	37	Evaluation of visuospatial/executive, naming, memory, attention, language, abstraction, delayed recall, and orientation. Telephone MoCA (T-MoCA) omits items about visuospatial/executive and naming
Mini-Mental State Examination (MMSE)	11	0–30	Interview	Face-to-face/Telephone	36	Evaluation of registration, attention, calculation, recall, language, ability to follow simple commands, and orientation. Several modified versions of MMSE are available for evaluation via telephone
Short Memory Questionnaire (SMQ)	14	4–46	Self-report	–	7	Evaluation of short-term memory, remote memory, cognition, orientation, and calculation. SMQ may also be evaluated by family caregivers
Mini-Cog	2	0–5	Interview	Face-to-face	1	Simple screening by the 3-item recall test and clock drawing test
Animal Naming	1	≥ 0	Interview	Face-to-face/Telephone	0	Simple screening by naming as many animals as possible within one minute
Repeatable Battery for the Assessment of Neuropsychological Status (RBANS)	12 (subtests)	≥ 0	Interview	Face-to-face	21	License fee required. Evaluation of immediate memory, visuospatial/constructional, attention, language, and delayed memory. It takes 30–45 min. RBANS has a mean (standard deviation) population age-adjusted score of 100 ± 15
Trail-Making Test A and B (TMT A and B)	2 (subtests)	≥ 0	Interview	Face-to-face	15	Evaluation of working memory, visual processing, visuospatial skills, selective and divided attention, processing speed, and psychomotor coordination. Measuring how long it takes to draw a line from the first circle to the 25th circle in a fixed order in each part
Mental health						
Hospital Anxiety and Depression Scale (HADS)	14	0–21	Self-report	–	155	Anxiety/depression
Impact of Event Scale-Revised (IES-R)	22	0–4 (average)	Self-report	–	76	PTSD
Impact of Event Scale 6 (IES-6)	6	0–4 (average)	Self-report	–	8	Short version of IES-R
Patient Health Questionnaire-4 (PHQ-4)	4	0–12	Self-report	–	3	Screening for anxiety and depression
Patient Health Questionnaire-9 (PHQ-9)	9	0–27	Self-report	–	26	Depression. PHQ-8 is also available that omits one item on suicidal ideation from PHQ-9
Generalized anxiety disorder-7 (GAD-7)	7	0–28	Self-report	–	13	Anxiety
Activities of daily living (ADL)						
Barthel Index	10	0–100	Self-report	–	47	Basic ADL including feeding, bathing, grooming, dressing, bowels, bladder, toilet, transfer, mobility, and stairs. The Barthel Index may also be evaluated by family caregivers
Instrumental Activities of Daily Living (IADL)	8	0–8	Self-report	–	25	Instrumental ADL including telephone, shopping, preparing food, housekeeping, laundry, transportation, medication, and finance. IADL may also be evaluated by family caregivers
Functional Independence Measure (FIM)	13	13–91	Interview	Face-to-face/Telephone	12	Evaluating not only basic ADL, but also communication and social problems. FIM may also be evaluated by family caregivers
Quality of life (QOL)						
Short Form-36 (SF-36)	36	0–100	Self-report	–	153	Usage fee required. SF-36 includes eight domains (physical functioning, role physical, bodily pain, general health, vitality, social functioning, role emotional, and mental health) and two component scores (physical component score [PCS] and mental component score]). The PCS score may be used for the assessment of physical problems. SF-36 may also be evaluated by family caregivers
European Quality of Life 5 Dimensions 5 Level, 3 Level, Visual Analogue Scale (EQ-5D-5L, 3L, VAS)	5	0–1	Self-report	–	155	Mobility, self-care, usual activities, pain/discomfort, and anxiety/depression components included. Components of mobility, self-care, and usual activities may be used for the screening of physical problems. The component of pain/discomfort may be used for the screening of pain. EQ-5D may also be evaluated by family caregivers using the proxy version
Short Form-12 (SF-12)	12	0–100	Self-report	–	26	Short form of SF-36, which also includes eight domains (physical functioning, role physical, bodily pain, general health, vitality, social functioning, role emotional, and mental health) and two component scores (physical component score [PCS] and mental component score]). Usage fee required. SF-12 may also be evaluated by family caregivers
12-item World Health Organization’s Disability Assessment Schedule (WHODAS 2.0)	12	0–48	Self-report		11	Evaluation of disability about mobility, self-care, life activities, and participation. WHODAS 2.0 may also be evaluated by family caregivers
Nutritional status						
Global Leadership Initiative on Malnutrition criteria (GLIM criteria)	3 (phenotype) 2 (etiology)	None Moderate Severe (malnutrition)	Interview	Face-to-face	–	Assessment to diagnose malnutrition among patients at risk. Phenotypic criteria include weight loss, a low body mass index, and reduced muscle mass. Etiological criteria include reduced food intake or assimilation and inflammation. To diagnose malnutrition, at least 1 phenotypic criterion and 1 etiological criterion need to be present. The severity of malnutrition is classified as moderate and severe
Body composition	–	–	Interview	Face-to-face	–	Evaluation of body composition including muscle mass by validated methods (e.g., dual-energy absorptiometry, bioelectrical impedance, ultrasound, computed tomography, or magnetic resonance imaging)
Sleep						
Pittsburgh Sleep Quality Index (PSQI)	19	0–21	Self-report	–	9	Sleep quality, latency, duration, efficiency, disturbance, medication, and daytime sleep dysfunction
Pain						
Brief Pain Inventory (BPI)	4 (severity) 7 (interference)	1–10	Self-report	–	13	Including two main scores: the pain severity score and pain interference score. BPI short form and long form are both available
Family						
SF-36	36	0–100	Self-report	–	47	QOL
HADS	14	0–14	Self-report	–	34	Anxiety/depression
IES-R	22	0–4 (average)	Self-report	–	19	PTSD

^a^We showed the frequency of articles on PICS conducted between 2014 and 2022, indicating the usage of each instrument [35]

The standard method for follow-up in the PICS clinic is face-to-face assessment, where we can use all the instruments listed in Table 1. An intensive care group in Germany proposed an approach starting from screening of PICS symptoms in outpatient settings [57]. For the screening, they recommended handgrip strength and the Timed Up-and-Go test for physical function, MiniCog and Animal Naming for cognition, PHQ-4 for mental health, and EQ-5D for health-related QOL. These instruments are very simple and highly sensitive, which are suited for screening. However, they exhibit relatively low specificity, necessitating extended evaluation. For example, the PHQ-4, with only four included items, has reported sensitivity and specificity of 0.90 and 0.61 for depression, and 0.88 and 0.61 for anxiety, respectively [58]. Alternatively, we can use other instruments that are slightly more time-consuming but offer greater specificity. These include 6-min walk test and MRC score for physical function, MoCA and MMSE for cognition, HADS and IES-R for mental health, and SF-36 for health-related QOL. These instruments have modest sensitivity and relatively high specificity. For example, HADS, with 14 included items, has reported sensitivity and specificity of 0.74 and 0.84 for depression, and 0.70 and 0.79 for anxiety, respectively [59, 60]. If these tools yield positive results, further evaluation should be considered by a multidisciplinary team, including professionals such as psychiatrists and physiotherapists. There is limited literature comparing diagnostic performance between instruments in patients with PICS. Therefore, the choice of instruments in the PICS clinic should align with the specific purpose and available resources.

PICS is also challenging for a patient’s family members and is assessed using PICS-family [61]. SF-36, HADS, and IES-R are recommended for the evaluation of PICS-family [35]. Furthermore, the concept of PICS was recently expanded from three main domains (physical, cognitive, and mental issues) to other domains, such as sleep disorders and chronic pain [7]. The Japanese Society of Intensive Care Medicine PICS committee has highlighted the importance of these insights, and recommends the Pittsburgh Sleep Quality Index for sleep disorders and Brief Pain Inventory for pain [35].

Nutrition therapy is one of the promising interventions for PICS after discharge. In the assessment of nutritional interventions, an international nutritional and metabolic group recommended the 30-s sit-to-stand test and the Global Leadership Initiative on Malnutrition (GLIM) for the nutrition status as essential components of the Outcome Set [55]. Furthermore, assessments of frailty and body composition are recommended, including an evaluation of muscle mass [62]. Frailty may be analyzed using the Clinical frailty scale (CFS) [63].

Muscle mass assessment as a body composition analysis is important in a PICS clinic to evaluate the nutritional status and physical function. Skeletal muscle mass in ICU survivors may be examined by validated methods, such as ultrasound or bioelectrical impedance analysis. Although dual-energy X-ray absorptiometry or CT can be used in the muscle mass assessment, these equipment and high cost hinder its routine use in PICS follow-up clinic [64, 65]. Ultrasound is non-invasive, but sufficient skills are needed for an accurate assessment [66]. A bioelectrical impedance analysis is non-invasive and not affected by inter-rater variability; however, fluid changes in some patients, may have an impact on the results obtained [67]. Muscle mass assessment is important, but there are still barriers to its implementation in PICS follow-up clinic. Particularly, it is difficult to implement in a telephone interview and online questionnaires, requiring future studies in this field.

### PICS assessment through a telephone interview and online questionnaires

Some ICU survivors cannot physically attend the PICS clinic and require follow-up through telephone interviews or online questionnaires. To evaluate these patients, we can use instruments available with telephone interview-style or self-report methods as listed in Table 1. The majority of instruments to assess mental health, QOL, and ADL may be used in a telephone interview, whereas physical and cognitive functions are often difficult to evaluate without visitation. Some instruments are available for telephone-based physical assessments. Health-related QOL may be examined through a telephone interview, and the physical component score of SF-36 or mobility, self-care, and usual activities of EQ-5D-5L are recommended for screening physical impairments as a part of health-related QOL [29, 55]. SF-12 (the short version of SF-36) is a useful instrument for calculating the physical component score of health-related QOL due to its simplicity [35]. Telephone-based ADL assessments including the Barthel Index may be useful for analyzing physical domains because ADL includes physical function as well as cognitive function. In previous studies, frailty was examined using CFS via a telephone interview [68, 69]. Since 40% of ICU survivors reported an increase in frailty after discharge [42], CFS may be useful to assess the physical domain [69].

Regarding cognitive functions, previous studies showed that a cognitive assessment through a telephone interview was not inferior to that in a face-to-face assessment [70, 71]. A telephone version of MoCA, termed T-MoCA, is conducted through a telephone interview [72]. This T-MoCA score may be changed into MoCA. MMSE also has a telephone version [73]. Self-reported methods are optional for an assessment of cognitive function, which may be evaluated through the telephone as well as online or in posted questionnaires. The Japanese Society of Intensive Care Medicine PICS committee recommends the Short Memory Questionnaire as a self-reported method for cognition, which may also be assessed by family caregivers [35]. In the absence of a dedicated PICS clinic, a telephone follow-up may be an option for ICU survivors.

## PICS treatment interventions

**Fig. 2:**
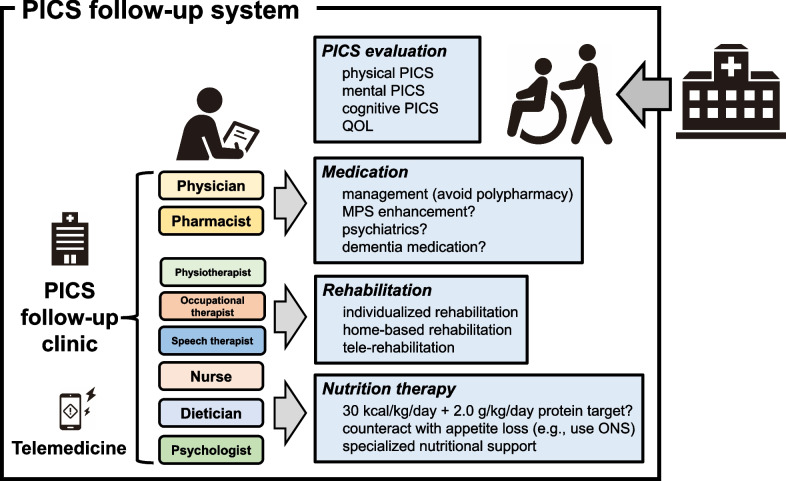
Interventions in the PICS follow-up system after discharge. Patients recovering from critical illness may receive interventions from medical staff in some PICS follow-up systems, in which the PICS follow-up clinic functions as the center. These systems may consist of a multi-disciplinary team for various approaches to PICS. They mainly provide patients with interventions involving medication, rehabilitation, and nutrition therapy based on a PICS evaluation in the same system

PICS treatment interventions mainly consist of medication, rehabilitation, and nutrition therapy. These interventions need to be provided by a multidisciplinary team (Fig. 2). Details on medication, rehabilitation, and nutrition therapy are summarized as follows.

### Medication approach

Two primary components are important for a medication approach: adjustments and interventions. Polypharmacy is common in critically ill patients due to the wide variety of symptoms [74]. Polypharmacy has been identified as a risk factor for readmission in ICU survivors [75]. Therefore, medication adjustments are needed after intensive care [76]. Furthermore, pharmacological interventions may be helpful for recovery from PICS [77]. Although some medications have been suggested to promote patient recovery, concrete evidence is insufficient [78].

A previous study revealed the prevalence of medication-related issues in PICS follow-up clinics. A PICS follow-up clinic survey in Scotland revealed that more than 60% of ICU survivors between 4 and 12 weeks after hospital discharge required interventions due to medication-related issues [79]. New medications started during the ICU stay are often the cause of intolerable side effects [80]. The most commonly discontinued and unnecessary medications in PICS follow-up clinics were proton pump inhibitors (19%), anticoagulants (12%), non-opioid pain analgesics (10%), and antipsychotics (7%) because they caused complications, such as an altered mental state, excessive sedation, and bleeding [50].

To achieve effective medication adjustments, the integration of pharmacists into a multidisciplinary follow-up team is a unique and invaluable contribution. Pharmacists may address drug interactions and adjustments for patients with impaired organ functions [81]. After ICU discharge, 9% of patients required an increased dose of or newly started sedatives or antipsychotics for their mental symptoms [50]. It is desirable for the same pharmacist to manage medications during and after ICU care because this provides a coherent approach from inpatient to outpatient settings [50]. A previous study reported that an intervention by pharmacists in a PICS follow-up clinic decreased the prevalence of medication-related issues [82].

Pharmacological interventions have been desired for some PICS components; however, there is insufficient evidence for its clinical application. The intervention to muscle protein synthesis, such as oxandrolone [78] or myostatin inhibitor [83], might enhance the recovery of physical impairments. A randomized controlled trial showed oxandrolone reduced muscle atrophy during the acute phase postburn [84], but the US Food and Drug Administration withdrew the medicine due to its complications (e.g., hepatitis, atherosclerosis, and malignancy) [85]. On the other hand, myostatin inhibitor modulated muscle atrophy and weakness [83], but the effect is not confirmed in clinical trials. Contrary to the physical impairments, few medications are effective for cognitive impairments and mental health even in the acute phase. Further researches are needed to develop medications for PICS treatment.

### Rehabilitation

**Fig. 3:**
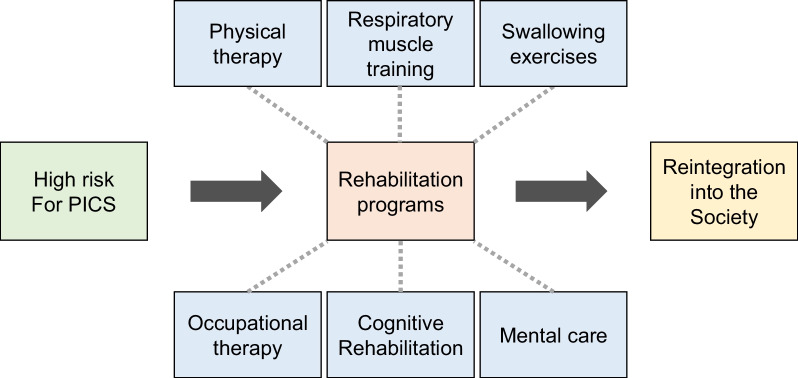
Rehabilitation programs from hospital discharge to reintegration into society. Rehabilitation programs include physical therapy, respiratory muscle training, swallowing exercises, occupational therapy, cognitive rehabilitation, and mental care

Rehabilitation after hospital discharge is crucial to aggressively regain functions for the reintegration of patients into their communities, in contrast to its preventive meaning in the acute phase of a critical illness. The urgency for effective post-discharge rehabilitation programs has grown in tandem with the increase in survival rates for various critical diseases in the ICU. Post-discharge rehabilitation comprises different types of interventions, including physical therapy, respiratory muscle training, swallowing exercises, occupational therapy, cognitive rehabilitation, and mental care (Fig. 3). Besides a multidisciplinary approach [86], the involvement of caregivers or family members has been shown to enhance the effects of the program through appropriate education and training as well as an understanding of their post-ICU life [87]. Education is important for successful adherence to rehabilitation [88, 89]. It provides essential support for patients, ensures their exercise adherence, and promptly identifies any complications.

A one-size-fits-all approach often falls short in addressing the needs of each patient [24]. Elliott et al. demonstrated that the application of a uniform physical rehabilitation program with five 1-h home visits at home and three telephone follow-ups for 8 weeks did not improve physical function [39]. In contrast, a 2-h rehabilitation program per week for the first 6 weeks, involving tailored supervision and education, increased walk distances [90]. The lack of a consensus on the intensity, frequency, or even contents of rehabilitation programs consistently emphasize the importance of personalized treatment plans based on thorough patient assessments in the follow-up period [91]. Individualized programs tailored for each patient represents the only approach to successfully meet individual needs and enhance both effectiveness and patient adherence, thereby leading to efficient and effective improvements in their post-ICU life [92, 93]. In this context, particularly for patients with no transportation methods or access to the facility, home-based rehabilitation has been reported to offer similar outcomes to institution-based programs [94]. These programs facilitate adherence by eliminating transportation barriers, providing familiar environments, and often having higher patient acceptance rates. In addition, the incorporation of the telerehabilitation system into these home-based programs allows for remote assessments, guidance, and monitoring by professionals, ensuring the continuity of care even when in-person sessions are not feasible [95]. These approaches may not only be cost-effective and expand access to specialized care for those in geographically distant areas, but also have the potential to provide rehabilitation programs after hospital discharge in future pandemics when isolation and strict infection regulations are in place.

### Nutrition therapy

Nutrition therapy is an essential support for the recovery of critically ill patients. From the viewpoint of muscle protein synthesis, adequate energy and protein provision is vital for maintaining and restoring muscle volume, which is linked to physical performance [96]. More nutritional support than usual is required after discharge as the convalescent period. As critical care nutrition guidelines have not addressed the details of nutritional support during this period [97, 98], there is lack of evidence regarding actual energy expenditure. Besides the perspective of disease complications and anabolic resistance, expert opinions indicate that 35 kcal/kg/day and 2.0–2.5 g/kg/day of protein are good targets during this period after discharge [99].

Nutritional intake after intensive care decreases to 30–50% of the optimal intake due to the end of enteral tube feeding [100–102]. This decreased nutritional intake continues after hospital discharge at 70% of the optimal nutritional intake even one year after discharge [103]. An insufficient nutritional intake was found to be dependent on the severity of prolonged physical impairments [104]. One of the main reasons for a decreased nutrition intake is a prolonged loss of appetite [105]. Abdominal swelling, nausea, vomiting, and tasting disorders contribute to a prolonged loss of appetite [102]. Prolonged depression and anxiety also decrease appetite [106]. Dysphagia, which occurs in 80% of ICU patients, persists in up to 60% of patients in the convalescent period [107, 108]. Therefore, malnutrition after ICU discharge is a serious issue that requires nutrition therapy interventions [53]. However, few studies have investigated nutritional interventions after hospital discharge. Salisbury et al. randomized patients with/without active rehabilitation, with continued nutritional support after discharge, but did not find any significant difference in physical outcomes [103]. However, calorie and protein intakes three months after discharge from the ICU were higher in the active rehabilitation group; 113.4% (71.9%–113.4%) vs. 70.0% (63.1%–95.9%) in energy and 90.3% (72.7%–126.1%) vs. 68.7% (61.9%–93.9%) in protein as percentages of estimated requirements. In this study, they primarily aimed at establishing the feasibility of these interventions and concluded that this type of post-discharge nutritional intervention was feasible.

Although there are currently no concrete recommendations for nutritional interventions, one possibility is the use of oral nutrition supplements. Since oral nutrition supplements are available in various tastes and smells with different amounts of protein, energy, and specific nutrients, dieticians may prescribe oral nutrition supplements based on a patient’s needs and preferences. Ridley et al. reported that patients receiving oral nutrition supplements had higher optimal energy and protein administration (73% and 68%, respectively) than those not receiving oral nutrition supplements (37% and 48%, respectively) [100]. Oral nutritional supplements need to be prescribed by dietitians to achieve optimal nutritional requirements [109]. There are currently no specific nutrient recommendations, including those for leucine, glutamine, arginine, carnitine, vitamin D, or ω3 fatty acids, due to the lack of significant findings and studies on PICS populations [98, 110]. β-Hydroxy β-methylbutyrate, a muscle protein synthesis stimulator, has potential as a nutritional intervention to gain muscle mass after discharge under a high protein supplement [111, 112].

## Future directions to treat PICS after discharge

Since PICS is an important social issue, health care workers need to monitor PICS after hospital discharge for its prevention [15]. However, the PICS follow-up system is not common. One of the important reasons for this is insufficient funding. Among facilities in the U.K., 90% did not receive funding and managed PICS follow-up clinics with own ICU budget [16]. There is currently no support for PICS follow-up clinics by the national health insurance systems in any country [41]. In the U.K., 90% of facilities without PICS follow-up clinics reported insufficient funding as the barrier [16]. In Japan, the Japanese Society of Intensive Care Medicine has now submitted a request to the Ministry of Health, Labour and Welfare, with the aim of creating national health insurance support for a PICS follow-up system. This type of financial support will accelerate PICS follow-ups.

Another barrier is insufficient evidence for the efficacy of the PICS follow-up system; therefore, patients cannot expect to receive benefits [16]. There are currently few intervention studies on the post-discharge phase, as discussed in this review. In contrast to the length of ICU stays, the duration after hospital discharge is sufficiently long to treat PICS. Therefore, a randomized controlled trial on interventions is needed. Due to the lack of adequate evidence, there are numerous possible interventions after hospital discharge, which include rehabilitation and nutritional interventions of different frequencies and degrees. Furthermore, the use of some drugs and patient care need to be investigated in future studies. PICS follow-up may play a leading role in this type of study.

The last barrier for the PICS follow-up system is the lack of recognition of PICS concept in not only patients and ICU workers, but also in the staffs in their hospitals and the other medical institutions outside hospitals. Awareness and education across and beyond each ICU and hospital have been argued as necessary from the PICS proposal era [1], nevertheless, the 2022 facility survey in our country revealed that the lack of understanding by the hospitals (71.8%), knowledges in ICU staffs (61.8%) and understandings by hospital administrator (53.6%) were raised as the barriers to perform PICS follow-up [23]. To promote the PICS follow-up system, we should return to the origin to expand much more awareness and education besides the financial support and evidences establishment.

Under the current widespread use of information technology, remote follow-up systems are expected to make a significant contribution to PICS follow-ups after hospital discharge. Besides conventional telephone interviews and mailed questionnaires, telemedicine is considered a new form of PICS follow-up clinic [113]. In a recent study, a telemedicine-through follow-up was well accepted by ICU survivors and their caregivers [45]. Telemedicine was also more readily accepted among pediatric populations and their families [114, 115]. The recent COVID-19 pandemic accelerated telemedicine technology, which has produced positive results [116, 117]. Some technologies require information technology literacy by both patients and health care workers, and future PICS follow-ups will require information technology-enabled ideas and flexibility.

## Conclusions

This review summarized the PICS follow-up system after hospital discharge and provides a comprehensive approach for the prevention and treatment of PICS. Although PICS assessments and follow-up methods vary, a multidisciplinary team approach is essential for the successful management of PICS.

## Data Availability

Not applicable.

## Abbreviations

ICU: Intensive care unit
PICS: Post-intensive care syndrome
QOL: Quality of life
MoCA: Montreal Cognitive Assessment
HADS: Hospital Anxiety and Depression Scale
PHQ-9: Patient Health Questionnaire-9
IES-R: Impact of Event Scale-Revised
SF-36: Short Form-36
EQ-5D: European Quality of Life 5 Dimensions
ADL: Activity of daily living
GLIM: Global Leadership Initiative on Malnutrition
CFS: Clinical frailty scale
COVID-19: Coronavirus disease 19
